# Differential gene expression response of synovial fibroblasts from temporomandibular joints and knee joints to dynamic tensile stress

**DOI:** 10.1007/s00056-021-00309-y

**Published:** 2021-06-17

**Authors:** Ute Nazet, Patrick Neubert, Valentin Schatz, Susanne Grässel, Peter Proff, Jonathan Jantsch, Agnes Schröder, Christian Kirschneck

**Affiliations:** 1 Department of Orthodontics, University Medical Centre of Regensburg, Regensburg, Germany; 2 Institute of Clinical Microbiology and Hygiene, University Hospital of Regensburg, Regensburg, Germany; 3 Department of Orthopaedic Surgery, Experimental Orthopaedics, Centre for Medical Biotechnology, University of Regensburg, Regensburg, Germany

**Keywords:** Osteoarthritis, Disease induction, Joint diseases, Animal model, Mechanical loading, Osteoarthrose, Entstehen einer Erkrankung, Gelenkerkrankungen, Tiermodell, Mechanische Belastung

## Abstract

**Purpose:**

Apart from other risk factors, mechanical stress on joints can promote the development of osteoarthritis (OA), which can also affect the temporomandibular joint (TMJ), resulting in cartilage degeneration and synovitis. Synovial fibroblasts (SF) play an important role in upkeeping joint homeostasis and OA pathogenesis, but mechanical stress as a risk factor might act differently depending on the type of joint. We thus investigated the relative impact of mechanical stress on the gene expression pattern of SF from TMJs and knee joints to provide new insights into OA pathogenesis.

**Methods:**

Primary SF isolated from TMJs and knee joints of mice were exposed to mechanical strain of varying magnitudes. Thereafter, the expression of marker genes of the extracellular matrix (ECM), inflammation and bone remodelling were analysed by quantitative real-time polymerase chain reaction (RT-qPCR).

**Results:**

SF from the knee joints showed increased expression of genes associated with ECM remodelling, inflammation and bone remodelling after mechanical loading, whereas TMJ-derived SF showed reduced expression of genes associated with inflammation and bone remodelling. SF from the TMJ differed from knee-derived SF with regard to expression of ECM, inflammatory and osteoclastogenesis-promoting marker genes during mechanical strain.

**Conclusions:**

Osteoarthritis-related ECM remodelling markers experience almost no changes in strain-induced gene expression, whereas inflammation and bone remodelling processes seem to differ depending on synovial fibroblast origin. Our data indicate that risk factors for the development and progression of osteoarthritis such as mechanical overuse have a different pathological impact in the TMJ compared to the knee joint.

**Supplementary Information:**

The online version of this article (10.1007/s00056-021-00309-y) contains supplementary material, which is available to authorized users.

## Abstract (de)

**Hintergrund:**

Mechanische Belastung von Gelenken kann abgesehen von anderen Risikofaktoren die Entwicklung von Osteoarthrose (OA) fördern. Dies kann auch das Kiefergelenk (TMJ) betreffen und zu Knorpeldegeneration und Synovitis führen. Synoviale Fibroblasten (SF) spielen eine wichtige Rolle bei der Aufrechterhaltung der Gelenkhomöostase und bei der OA-Pathogenese, aber je nach Art des Gelenks kann sich mechanische Belastung als Risikofaktor unterschiedlich auswirken. Wir untersuchten daher den relativen Einfluss von mechanischem Stress auf das Genexpressionsmuster von SF aus dem Kiefergelenk und dem Kniegelenk, um neue Erkenntnisse zur OA-Pathogenese zu gewinnen.

**Methoden:**

Aus Kiefer- und Kniegelenken von Mäusen isolierte primäre SF wurden mechanischer Belastung unterschiedlichen Ausmaßes ausgesetzt. Dabei wurde mittels RT-qPCR („quantitative real-time polymerase chain reaction“) die Expression von Markergenen der extrazellulären Matrix (ECM), der Entzündung und des Knochenumbaus analysiert.

**Ergebnisse:**

In SF aus den Kniegelenken war nach mechanischer Belastung die Expression von Genen, welche mit ECM-Umbau, Entzündung und Knochenumbau assoziiert sind, erhöht, während in aus Kiefergelenken isolierten SF die Expression von in Entzündung und Knochenumbauprozesse involvierten Genen reduziert war. Des Weiteren unterschieden sich während der mechanischen Belastung TMJ-SF und Kniegelenk-SF hinsichtlich der Expression von Markergenen für ECM, Entzündung und Förderung der Osteoklastogenese.

**Schlussfolgerungen:**

Osteoarthrosebezogene ECM-Remodellierungsmarker erfahren fast keine Veränderungen in der belastungsinduzierten Genexpression, wohingegen Entzündungs- und Knochenumbauprozesse in Abhängigkeit von der Herkunft der SF unterschiedlich beeinflusst zu sein scheinen. Unsere Daten deuten darauf hin, dass Risikofaktoren für die Entwicklung einer OA und deren Fortschreiten, wie z. B. mechanische Überbeanspruchung, im TMJ einen anderen pathologischen Einfluss haben als im Kniegelenk.

## Introduction

Full movement and flexibility of the human body is enabled by various types of joints. Their structural composition, however, is very specific. There are synovial joints imbedded in an articular capsule interconnecting bones as well as fibrous and cartilaginous joints, characterised by their connective tissue appearance [34]. Joint disorders such as osteoarthritis impede joint functionality of major joints within the human body, including the temporomandibular joint (TMJ), thus affecting quality of life as well as performance effectiveness [22, 29]. Osteoarthritis affects subjects with increasing age, joints exposed to mechanical stress and depends on dietary food intake and factors of metabolic and genetic origin [13, 18, 19, 27]. As osteoarthritis diminishes effective performance ability and quality of life, research in this topic promises pain relief as well as the determination of the impact of various factors on osteoarthritis development and progression.

For several years a variety of treatment options promising patients pain relief and improvement of joint functionality have been established. Apart from general recommendations, such as lifestyle changes, also pain-relieving medication (e.g. non-steroidal anti-inflammatory drugs) and supportive treatments (e.g. transcutaneous electrical nerve stimulation, physiotherapist-guided manual therapy or assistive devices) can reduce pain. Another noninvasive alternative treatment option is laser therapy and low intensity pulsed ultrasound, as they promise pain relief, while patients experience few side effects [2, 9, 31, 44]. Furthermore in vivo OA-model studies indicate favourable effects by the application of ultrasound treatment, concerning cartilage repair, extracellular matrix destruction and vascularisation [15, 16, 46]. Surgical treatment options are considered if noninvasive options show no effect and the joint is severely damaged. In these cases, minimally invasive procedures can be performed to remove scar tissue, reshape the joint or relieve swelling, while removing inflammatory byproducts. In case of open surgery, total joint replacement can be performed as well as joint fusing and osteotomy.

In general, osteoarthritis is an illness based on a misbalanced interplay between several cell populations located in the joints [6, 55]. This imbalance supports cartilage degradation, osteophyte formation, subchondral bone reduction and synovial inflammation. Currently, the role of the synovial membrane in osteoarthritis development and progression is being investigated with increasing interest. Synovial fibroblasts, which constitute the major cell population of the synovial membrane, are involved in joint lubrication, tissue homeostasis and nourishment [7, 14, 30]. A clinical indicator of osteoarthritis among others is synovitis associated with a thickened and inflammatory active synovial membrane. During inflammation and osteoarthritis, the synovial membrane undergoes changes causing enhancement and induction of inflammation, altered joint lubrication composition and a signalling shift in bone remodelling [48]. Apart from research on human primary cells, derived from osteoarthritis-affected patients and patients without osteoarthritis, research focuses on model-organism-derived primary cells. Hence, major research regarding osteoarthritis development and progression is performed on knee joints. But also the TMJ can be affected by arthritic changes with a high impact on quality of life. Beside the fact that both joints belong to the family of synovial joints, they differ in their embryonic development.

During embryogenesis, the structures of the knee joint develop from the outer and central mesenchyme, forming the joint capsule, ligaments and synovial cavities [35]. In contrast, the TMJ emerges from the ectoderm being guided by Hedgehog signalling pathways, while the secondary cartilage develops [28]. Apart from differences in development, both joints also differ in their mode of operation. In the knee joint, movement is only possible in a single axis, defined by fixating tendons, muscles and structural components, categorizing it as a member of the hinge-joint family [34]. In contrast, the TMJ is a hinge and sliding joint, as the interplay between the disc, the glenoid fossa of the temporal bone and the connective tissue enables joint articulation on a single plane and axis [1, 51]. These differences in mechanical and structural composition (single axis movement versus single plane and axial movement), as well as embryogenic development may have an impact on the factors that induce osteoarthritis. Therefore, we analysed the differential effects of different mechanical stimuli in the context of osteoarthritic inflammation of the knee and TMJ joint on extracellular matrix composition and bone remodelling in synovial fibroblasts derived from temporomandibular joints and compared the findings to data derived from knee joints.

## Material and methods

### Isolation of synovial fibroblasts

**Fig. 1:**
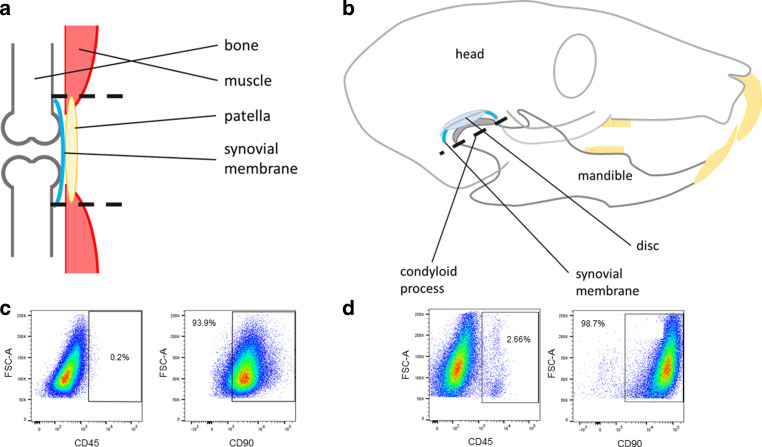
Isolation and characterisation of murine synovial fibroblasts (SF) derived from the knee and temporomandibular joint (TMJ). After removal of superficial tissue, the joint capsule of the knee (**a**) was opened to dissect the synovial membrane, while in the capsule of the mandibular joint (**b**) the synovial tissue of the TMJ was located. After several days, outgrowing of spindle-shaped cells was observed from TMJ samples and knee samples. The proportion of cells positive for leukocyte-specific antigens (CD45) and fibroblast-specific antigens (CD90) was determined via fluorescence-activated cell sorting (FACS) analysis (**c** primary knee SF; **d** primary TMJ SF) Isolierung und Charakterisierung von murinen synovialen Fibroblasten (SF) aus dem Knie- und dem Kiefergelenk (TMJ). Nach Entfernung des oberflächlichen Gewebes wurde die Gelenkkapsel des Knies (**a**) geöffnet, um die Synovialmembran zu sezieren, während in der Kapsel des TMJ (**b**) das entsprechende synoviale Gewebe zu finden war. Nach einigen Tagen wurde sowohl in den TMJ- als auch in den Kniegelenkproben ein Auswachsen spindelförmiger Zellen beobachtet. Der Anteil an für leukozytenspezifische (CD45) und fibroblastenspezifische (CD90) Antigene positiven Zellen wurde mittels FACS(„fluorescence-activated cell sorting“)-Analyse bestimmt (**c** primäre Kniegelenk-SF, **d **primäre TMJ-SF)

For isolation of synovial fibroblasts, wildtype BL/6 mice (male, age: 9 weeks) were killed and dissected conforming to national and institutional regulations. The knee capsule was opened by radial cuts above the patella, and the patella with attached synovial tissue was harvested in ice-cold Dulbecco’s modified Eagle’s medium (DMEM; D5671, Sigma Aldrich, St. Louis, MO, USA), supplemented with 1% L‑glutamine solution (L-Glut; G7513, Sigma Aldrich, St. Louis, MO, USA) and 1% antibiotic antimycotic (AA; A5955, Sigma Aldrich, St. Louis, MO, USA; Fig. 1a). For isolation of the temporomandibular synovial tissue, first the superficial masseter muscle was removed, providing open access to the condyloid process located in the socket of the mandibular fossa beneath the zygomatic process of the temporal bone. The condyloid process was lifted and the condylar head including the articular disc and attached synovial tissue was extracted and transferred to ice-cold DMEM (Fig. 1b). For all dissection steps, a Nikon SMZ 1500 microscope (Nikon, Minato, Tokyo, Japan) was used. After dissection, the harvested tissues were washed twice with Dulbecco’s phosphate buffered saline (PBS; 14190-094, Gibco, Thermo Fisher Scientific, Waltham, MA, USA), supplemented with 1% antibiotic antimycotic in a sterile laminar airflow cabinet. For digestion, the respective bilateral knee or TMJ tissue of five animals was pooled (10 knees or 10 TMJs), cut into small pieces and transferred to DMEM (supplemented with 10% fetal bovine serum (FBS; P30-3302, Pan Biotech, Aidenbach, Germany), 1% L‑Glut, 1% AA and 0.1% collagenase IV (C4-28, Biochrom, Merck Millipore, Burlington, MA, USA)) for at least 2 h at 37 °C [3]. To improve digestion, the samples were mixed several times during incubation. After incubation, the samples were centrifuged and the pellet was resuspended in fresh DMEM (supplemented with 10% FBS, 1% AA, 1% L‑Glut and 1% ascorbic acid). Tissue pieces and floating cells were plated in separate cell culture containers, which had been coated the day before with collagen I (ALX-522-435-0020, Enzo Life Sciences, Lörrach, Germany). After several days, outgrowing cells with spindle-shape appearance could be observed.

### Characterisation of synovial fibroblasts

For characterisation of harvested cells, the presence of fibroblastic antigens (CD90, dilution 1:100) and absence of leukocytic antigens (CD45, dilution 1:100; Fig. 1c, b) was determined [3, 42].

Gene expression was verified by semiqantiative PCR analysis. RNA was harvested and cDNA was generated, followed by PCR analysis: 2 ng cDNA were supplemented with 0.25 µl 10 pmol forward primer, 0.25 µl 10 pmol reverse primer (Supplemental Table 1), 1 µl 10 × buffer + MgCl_2_ (14800100; Roche), 0.2 µl 200 µM dNTPs (L785.1/2; Carl-Roth), 0.1 µl Taq-Pol (39469200; Roche) and nuclease-free water up to a final volume of 10 µl. After initiation (95 °C, 5 min), 40 cycles of ligation and elongation (95 °C for 20 s, 65 °C for 30 s) were applied. Finally, products were stored at 4 °C until further application. Product analysis was done on a 1% agarose 1 × TAE gel (Tris-acetate-EDTA; 50 × TAE: 2 M Tris, 50 mM EDTA, 1 M glacial/acetic acid; pH 8.3; supplemental Fig. 1) [26, 54].

Antigen presence was determined by FACS (fluorescence-activated cell sorting; BD FACS Canto™ II, BD Biosciences, San Jose, CA, USA) analysis. To this aim, 150,000 cells were harvested, washed and resuspended in 50 µl 1 × phosphate-buffered saline, supplemented with 1 µl FC-Block (101319, Biolegend, San Diego, CA, USA) and incubated for 5 min at 4 °C. Subsequently, 1 µl of the antibodies of interest was added (CD90, 105327, Biolegend, San Diego, CA, USA; CD45, 109831, Biolegend, San Diego, CA, USA) and the cells were incubated for a further 30 min at 4 °C. After incubation, 1 ml PBS was added and the cell suspension was centrifuged (5 min, 300 g, 4 °C). After discarding the supernatant, the pellet was resuspended in 100 µl PBS and immediately analysed via FACS. All steps were performed in dark conditions.

### Dynamic stretching protocols

**Fig. 2:**
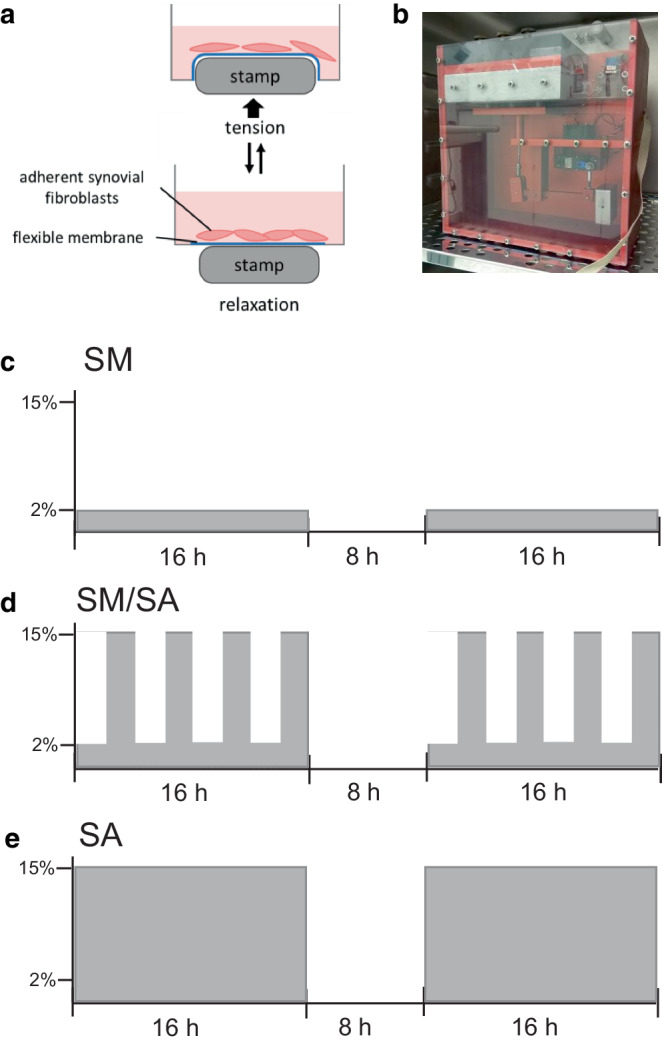
Visualization of applied dynamic stretching protocols (**a**) performed in a custom made cell stretching device (**b**) composed of distinct protocols of moderate stretching (SM; **c**), mixed stretching (SM/SA; **d**) and advanced stretching (SA; **e**) Visualisierung der angewandten dynamischen Dehnungsprotokolle (**a**), durchgeführt in einem speziell angefertigten Zelldehnungsgerät (**b**). Die Dehnungsprotokolle bestanden aus Protokollen für moderate (SM; **c**), gemischte (SM/SA; **d**) und anspruchsvolle Dehnung (SA; **e**)

The experimental dynamic stretching assay consisted of three distinct stretching protocols, containing two identical blocks of stretching [37, 41]. After preincubating 70,000 synovial fibroblasts per well on a collagen-I-coated 6‑well plate (BF-3001C, Flexcell, Burlington, NC, USA) for 24 h, the first block of 16 h of stretching was applied, followed by a break of 8 h and a second block of stretching (16 h), which was also the final one. In the moderate stretching protocol (SM; Fig. 2a), cells were stretched at a frequency of 0.2 Hz and an amplitude of 2% per block. In the advanced stretching protocol (SA; Fig. 2c), cells were stretched at a frequency of 0.5 Hz and an amplitude of 15%, while in the mixed stretching protocol (SM/SA; Fig. 2b), cells were stretched at a frequency of 0.2 Hz and 2% for 2 h and 0.5 Hz and 15% for 2 h in continuous alternation per block. For stretching, a custom-made cell-stretching machine was used, offering a 6-well plate fitting slot and 6 stamps, capable to simultaneously elongate according to a previously compiled stretching protocol (Fig. 2). For the experiments, cells of passages 6 and 7 were used.

### Determination of cell number

After dynamic stretching, synovial fibroblasts were scrapped off the cell culture plate and resuspended in 1 ml PBS. For determination of cell number, 100 µl of the cell suspension were mixed with 10 ml 0.9% saline and cell number was immediately measured with a Beckman Coulter Counter Z2 (Beckman Coulter GmbH, Krefeld, Germany).

### Determination LDH release

LDH (Lactate Dehydrogenase Activity) assay was performed following the manufacturer’s instructions (04744926001, Roche) using the cell culture supernatant after dynamic stretching protocols. Briefly, cell culture supernatant was mixed with LDH solution (22 µl catalyst with 1 ml dye) 1:1 and incubated for 30 min at room temperature in the dark. After stopping the reaction by adding stop solution, the absorbance at 490 nm was measured, using a fluorescent plate reader (Multiscan GO Microplate Spectrophotometer, Thermo Fisher Scientific, Waltham, MA, USA). Background absorbance at 690 nm was subtracted.

### RNA isolation

We isolated RNA using peqGOLD TriFast^TM^ (30-2020, Peqlab, VWR, Radnor, PA, USA). Briefly, 500 µl peqGOLD TriFast^TM^ was added per sample and vortexed after addition of 100 µl chloroform (102445, Merck, Darmstadt, Germany) for at least 20 s. Approximately 200 µl colourless supernatant was transferred to a tube with 500 µl precooled isopropanol (20.842.330, VWR, Radnor, PA, USA) and incubated overnight at −80 °C. Samples were centrifuged for 30 min at 4 °C 13,000 rpm and the obtained pellet was washed twice with 80% ethanol (32205-1L‑M, Sigma-Aldrich, St. Louis, MO, USA). After the last washing step, the pellet was dried for at least 30 min and RNA concentration was determined using a NanoPhotometer (N60, Implen, Munich, Germany) after reconstitution in 20 µl RNase-free water (T143, Carl Roth, Karlsruhe, Germany).

### cDNA synthesis

To reduce experimental variation, reverse transcription reagents were used as master mix. For one sample, we mixed 1 µl oligo-dT18 primer (SO131, Thermo Fisher Scientific, Waltham, MA, USA), 1 µl random hexamer primer (SO142, Thermo Fisher Scientific, Waltham, MA, USA), 1 µl dNTP mix (L785.2, Carl Roth), 1 µl RNase inhibitor (EO0381, Thermo Fisher Scientific, Waltham, MA, USA), 1 µl MLV-reverse transcriptase (M1705, Promega, Madison, WI, USA) and 4 µl 5 × M-MLV-buffer (M1705, Promega). Equal amounts of RNA (50 ng) were diluted in a total volume of 5.5 µl and mixed with 4.5 µl prepared master mix (total volume of cDNA reaction: 10 µl). RNA was transcribed within 60 min at 37 °C followed by 2 min at 95 °C. Finally, the cDNA was diluted with RNAse-free water (T143, Carl Roth, Karlsruhe, Germany) to a final concentration of 1 ng/µl for storage and further semiquantitative and quantitative real-time PCR performance.

### Quantitative real-time polymerase chain reaction

To reduce experimental variation, a master mix of quantitative real-time polymerase chain reaction (RT-qPCR) reagents was used. For each sample, we mixed 7.5 µl SYBR®Green JumpStart Taq ReadyMix (S4438, Sigma Aldrich, St. Louis, MO, USA), 0.75 µl forward and 0.75 µl reverse primer with 5.25 µl RNAse-free water (T143, Carl Roth). Per sample we applied 13.5 µl per well of a 96-well PCR plate (TW-MT, 712282, Biozym Scientific, Hessisch Oldendorf, Germany), mixed it with 1.5 µl cDNA (1 ng/µl) and sealed the plate with a cover (712350, Biozym Scientific, Hessisch Oldendorf, Germany). After initial heating for 5 min at 95 °C, RT-qPCR was performed in 45 cycles (each cycle: 10 s at 95 °C, 8 s at 60 °C, 8 s at 72 °C; Mastercycler® ep realplex‑S thermocycler, Eppendorf AG, Hamburg, Germany). We calculated relative gene expression using a set of two reference genes which proved to be suitable for the used experimental setup based on the 2^−∆Cq^ method (∆Cq = Cq (target gene) − Cq (geometric mean *Hprt/Sdha*)) [49, 50]. All primers (Table 1; Eurofins MWG, Ebersberg, Germany) were constructed with NCBI PrimerBLAST [10]. *Hprt* and *Sdha* were used as reference genes, as they showed the most stable expression in the experimental setup (unpublished data). For RT-qPCR analysis two technical and at least 3–5 biological replicates and three repetitions per stretching protocol run were performed. The data are presented as fold-change in comparison to the mean gene expression detected in the control group (unstretched cells).

**Table 1:** Quantitative real-time polymerase chain reaction (RT-qPCR) primer sequences for reference genes (*Hprt, Sdha*) and target genes RT-qPCR(„quantitative real-time polymerase chain reaction“)-Primersequenzen für die Referenzgene (*Hprt, Sdha*) und Zielgene

Gene symbol	Gene name	5’-forward primer-3’	5’-reverse primer-3’	NCBI GeneBank accession number	Amplicon size, T_m_, GC%
*Adamts5*	A disintegrin and metalloproteinase with thrombospondin motifs 5	ACAAGTGTGGAGTGTGCGGAG	ATGTGGGTTGCTCCTTCAGGG	NM_011782.2	118 bp, 62 °C, 57
*Cemip2*	Cell migration inducing hyaluronidase 2	AGCCGTTCTCAGGTCAAAGTC	TTGCGGGTGAGAAGTCCAAC	NM_001033759.2	104 bp, 63 °C, 52–55
*Col1a1*	Collagen, type I, alpha 1	CAGAGGCGAAGGCAACAGTC	TGACTGTCTTGCCCCAAGTTC	NM_007742.4	86 bp, 63 °C, 60–52
*Col1a2*	Collagen, type I, alpha 2	TGGCCCCAATGGATTTGCTG	CCTTAGGCCCTTTGGTTCCC	NM_007743.3	83 bp, 61 °C, 55–60
*Fn1*	Fibronectin 1	TGCTCAACCCACTCCCGATG	CGGCATGAAGCACTCAATGGG	NM_001276412.1	112 bp, 62 °C, 60–57
*Has1*	Hyaluronan synthase 1	TGACAGGCACCTCACCAACC	TGGCTCAACCAACGAAGGAAGG	NM_008215.2	111 bp, 63 °C, 60–54
*Hprt*	Hypoxanthine guanine phosphoribosyl transferase	AGCTTGCTGGTGAAAAGGAC	AGTCAAGGGCATATCCAACAAC	NM_013556.2	100 bp, 58 °C, 50-45
*Il1β*	Interleukin 1 beta	GTGTAATGAAAGACGGCACACC	ACCAGTTGGGGAACTCTGC	NM_008361.4	150 bp, 60 °C, 50–57
*Il1-ra*	Interleukin 1 receptor antagonist	GCTCATTGCTGGGTACTTACAA	CCAGACTTGGCACAAGACAGG	NM_031167.5	132 bp, 63 °C, 45–57
*Il6*	Interleukin 6	ACAAAGCCAGAGTCCTTCAGAG	GAGCATTGGAAATTGGGGTAGG	NM_031168.2	108 bp, 60 °C, 50
*Opg*	Osteoprotegerin	CCTTGCCCTGACCACTCTTAT	CACACACTCGGTTGTGGGT	NM_008764.3	127 bp, 60 °C, 52–57
*Ptgs2*	Prostaglandin-endoperoxide synthase 2	TCCCTGAAGCCGTACACATC	TCCCCAAAGATAGCATCTGGAC	NM_011198.4	149 bp, 59 °C, 55–50
*Rankl*	Tumour necrosis factor superfamily, member 11	AAACGCAGATTTGCAGGACTC	CCCCACAATGTGTTGCAGTTC	NM_011613.3	118 bp, 60 °C, 47–52
*Sdha*	Succinate dehydrogenase complex, subunit A, flavoprotein	AACACTGGAGGAAGCACACC	AGTAGGAGCGGATAGCAGGAG	NM_023281.1	135 bp, 60 °C, 55–57

### Statistical analysis

GraphPad Prism version 8.4 for Windows (GraphPad Software, San Diego, CA, USA) was used for statistical analysis. Mixed-effects analysis with Geisser–Greenhouse correction followed by Holm–Sidak’s multiple comparison tests was performed to determine differences between knee- and TMJ-derived synovial fibroblasts and Dunett’s multiple comparison tests to assess differences between different stretching protocols. Significance level was set at *p* < 0.05.

## Results

### Synovial fibroblast origin and dynamic stretching

#### Impact on cell number and cytotoxicity

**Fig. 3:**
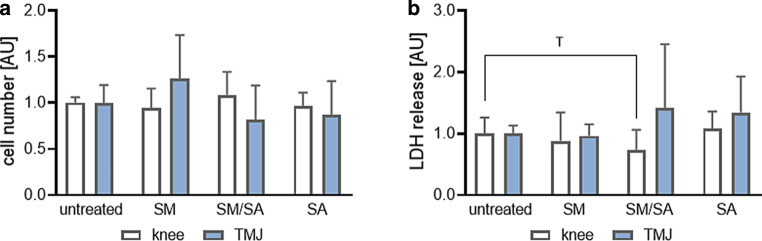
Effects of dynamic stretching protocols on cell number (*n*_knee_ = 13, *n*_TMJ_ = 11; **a**) and cytotoxicity (*n*_knee_ = 13, *n*_TMJ_ = 12; **b**) in synovial fibroblasts derived from knee and temporomandibular joint after different dynamic loading protocols. *AU* arbitrary unit, *SM* moderate, *SM/SA* intermediate, *SA* advanced. Statistics: Mixed-effects analysis with Geisser–Greenhouse correction followed by Holm–Sidak’s multiple comparisons tests. ^T^*p* < 0.10 Auswirkungen der dynamischen Dehnungsprotokolle auf die Zellzahl (*n*_Knie_ = 13, *n*_TMJ_ = 11; **a**) und Zytotoxizität (*n*_Knie_ = 13, *n*_TMJ_ = 12; **b**) in synovialen Fibroblasten aus Knie- und Kiefergelenk nach unterschiedlichen dynamischen Belastungsprotokollen. *AU* arbiträre Einheit, *SM* moderat, *SM/SA* gemischt, *SA* anspruchsvoll. Statistik: Mixed-effects-Analyse mit Geisser-Greenhouse-Korrektur, gefolgt von Holm-Sidaks „multiple comparisons tests“. ^T^*p* < 0,10

First, we investigated the effects of dynamic stretching on synovial fibroblast number. Cell number was not affected by any tested dynamic stretching protocol in knee- (SM: *p* = 0.6441; SM/SA: *p* = 0.4945; SA: *p* = 0.5958) and TMJ-derived synovial fibroblasts (SM: *p* = 0.2314; SM/SA: *p* = 0.3650; SA: *p* = 0.5640; Fig. 3a). Accordingly, we detected no differences in number of fibroblasts from the knee or the TMJ (SM: *p* = 0.2031; SM/SA: *p* = 0.2031; SA: *p* = 0.6720). In line with cell number, LDH release was not affected by the various dynamic stretching protocols in synovial fibroblasts of knee (SM: *p* = 0.6441; SM/SA: *p* = 0.4945; SA: *p* = 0.5958) and TMJ origin (SM: *p* = 0.6441; SM/SA: *p* = 0.4945; SA: *p* = 0.5958; Fig. 3b), indicating no cytotoxic effects of the dynamic stretching protocols applied.

#### Impact on extracellular-matrix-remodelling gene expression

**Fig. 4:**
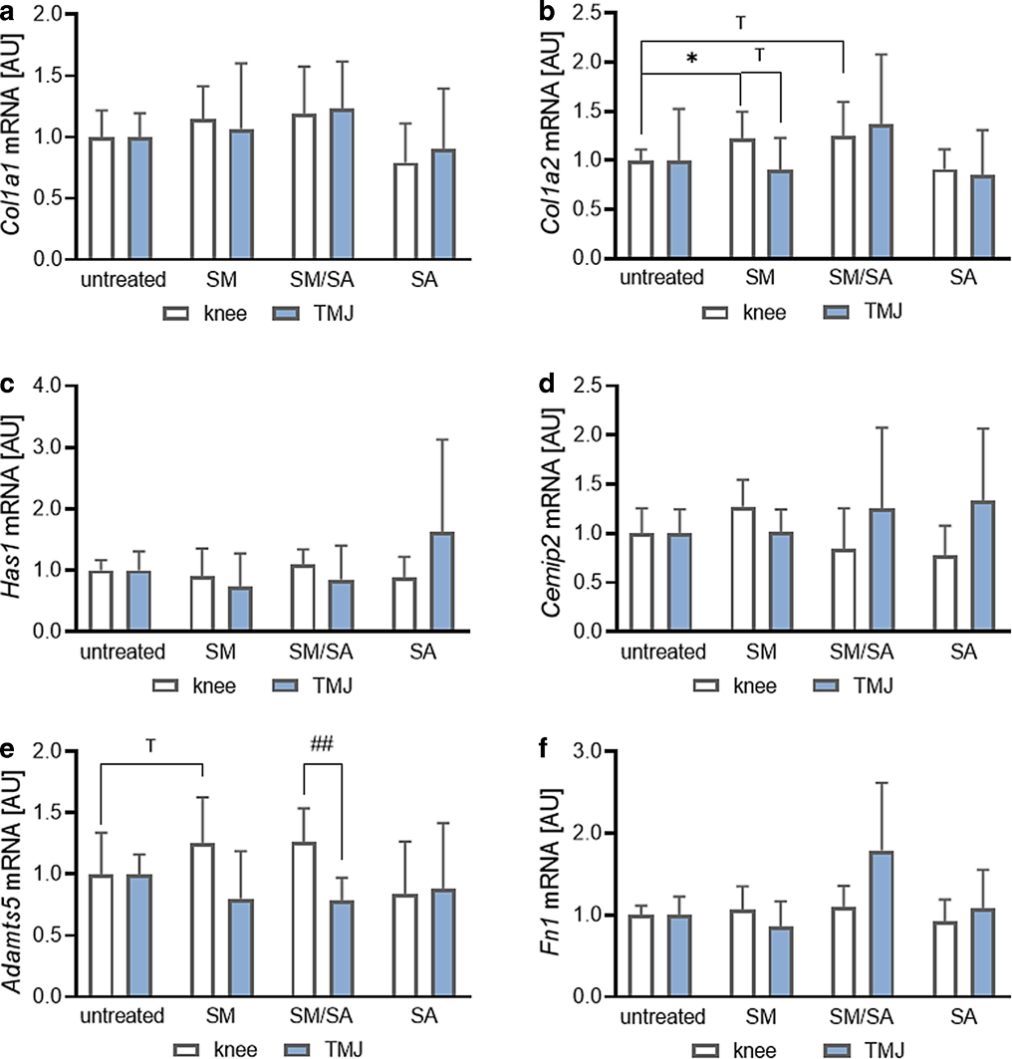
Effects of dynamic stretching protocols on the genes *Col1a1* (*n*_knee_ = 13, *n*_TMJ_ = 10; **a**), *Col1a2* (*n*_knee_ = 12, *n*_TMJ_ = 11; **b**), *Has1* (*n*_knee_ = 12, *n*_TMJ_ = 11; **c**), *Cemip2* (*n*_knee_ = 8, *n*_TMJ_ = 10; **d**), *Adamts5* (*n*_knee_ = 8, *n*_TMJ_ = 6; **e**) and *Fn1* (*n*_knee_ = 8, *n*_TMJ_ = 6; **f**) involved in extracellular-matrix remodelling in synovial fibroblasts derived from the knee and temporomandibular joint (TMJ). *AU* arbitrary unit, *SM* moderate, *SM/SA* intermediate, *SA* advanced. Statistics: Mixed-effects analysis with Geisser–Greenhouse correction followed by Holm–Sidak’s multiple comparisons tests. ^T^*p* < 0.10, *p < 0.05 Effekte der dynamischen Dehnungsprotokolle auf die Gene *Col1a1* (*n*_Knie_ = 13, *n*_TMJ_ = 10; **a**), *Col1a2* (*n*_Knie_ = 12, *n*_TMJ_ = 11; **b**), Has1 (*n*_Knie_ = 12, *n*_TMJ_ = 11; **c**), *Cemip2* (*n*_Knie_ = 8, *n*_TMJ_ = 10; **d**), *Adamts5* (*n*_Knie_ = 8, *n*_TMJ_ = 6; **e**) und *Fn1* (*n*_Knie_ = 8, *n*_TMJ_ = 6; **f**), die am Umbau der extrazellulären Matrix in synovialen Fibroblasten aus dem Knie- und dem Kiefergelenk (TMJ) beteiligt sind. *AU* arbiträre Einheit, *SM* moderat, *SM/SA* gemischt, *SA* anspruchsvoll. Statistik: Mixed-effects-Analyse mit Geisser-Greenhouse-Korrektur, gefolgt von Holm-Sidaks „multiple comparisons tests“. ^T^*p* < 0,10, ^*^p < 0,05

The extracellular matrix consists of fibrous components, various glycoproteins and polysaccharides. The predominant protein family are collagens, which mainly consist of alpha‑1 and alpha‑2 polypeptide chains. The second largest group are glycosaminoglycans, which can interact with proteins to form proteoglycans. Gene expression of *collagen-1-alpha‑1* (*Col1a1*) was not affected by any dynamic stretching protocol applied on knee- (SM: *p* = 0.2796; SM/SA: *p* = 0.3042; SA: *p* = 0.2317) and TMJ-derived synovial fibroblasts (SM: *p* = 0.9630; SM/SA: *p* = 0.3149; SA: *p* = 0.8958; Fig. 4a). In contrast, *collagen-1-alpha‑2 (Col1a2)* gene expression was elevated in knee-derived synovial fibroblasts after moderate stretching (SM: *p* = 0.0352; Fig. 4b). The mixed stretching protocol tended to increase *Col1a2* gene expression (SM/SA: *p* = 0.0762), while advanced stretching had no effect on *Col1a2* gene expression in knee-derived synovial fibroblasts (SA: *p* = 0.3926; Fig. 4b). Synovial fibroblasts derived from the TMJ failed to change gene expression of *Col1a2* upon any tested stretching protocol (SM: *p* = 0.9350; SM/SA: *p* = 0.5057; SA: *p* = 0.7993; Fig. 4b). Therefore *Col1a2* tended to be differentially expressed after moderate stretching in knee- and TMJ-derived synovial fibroblasts (SM: *p* = 0.0629). Hyaluronic acid is a polysaccharide synthesized by membrane-bound hyaluronic acid synthases (Has). Gene expression of *Has1* was not affected by any tested stretching protocol in knee- (SM: *p* = 0.8696; SM/SA: *p* = 0.5254; SA: *p* = 0.6385) and TMJ-derived synovial fibroblasts (SM: *p* = 0.4618; SM/SA: *p* = 0.8149; SA: *p* = 0.4153; Fig. 4c). Accordingly, gene expression of cell-migration-inducing hyaluronidase 2 (*Cemip2*), which cleaves extracellular high molecular weight hyaluronan, was not affected in knee- (SM: *p* = 0.1395; SM/SA: *p* = 0.6544; SA: *p* = 0.4198) and TMJ-derived synovial fibroblasts by stretching (SM: *p* = 0.9982; SM/SA: *p* = 0.7111; SA: *p* = 0.3437; Fig. 4d). *Adamts5* encodes for a member of a disintegrin and metalloproteinase protein family with thrombospondin motifs, which may be involved in osteoarthritis. In knee-derived synovial fibroblasts, *Adamts5* gene expression tended to be upregulated with moderate stretching (SM: *p* = 0.0765), while mixed and advanced stretching did not impact *Adamts5* gene expression (SM/SA: *p* = 0.4071; SA: *p* = 0.8062; Fig. 4e). In contrast, in TMJ-derived synovial fibroblasts *Adamts5 *gene expression was not affected by any stretching protocol tested (SM: *p* = 0.4844; SM/SA: *p* = 0.2580; SA: *p* = 0.8414), therefore resulting in significant differences between knee- and TMJ-derived synovial fibroblasts in *Adamts5* expression with the mixed stretching protocol (SM/SA: *p* = 0.0085; Fig. 4e). Last we analysed gene expression of *Fibronectin‑1* (*Fn1*). Fibronectin is a glycoprotein of the extracellular matrix and altered expression has been associated with a number of pathologies. Neither knee- (SM: *p* = 0.8086; SM/SA: *p* = 0.6890; SA: *p* = 0.8318) nor TMJ-derived (SM: *p* = 0.6612; SM/SA: *p* = 0.1876; SA: *p* = 0.9337) synovial fibroblasts changed *Fn1* gene expression upon any tested stretching protocol with no differences depending on origin (Fig. 4f). From that data we conclude that mechanical loading impacted only knee-derived synovial fibroblasts on *Col1a2* and *Adamts5* gene expression, while TMJ-derived synovial fibroblasts failed to react to mechanical loading.

#### Impact on inflammatory gene expression

**Fig. 5:**
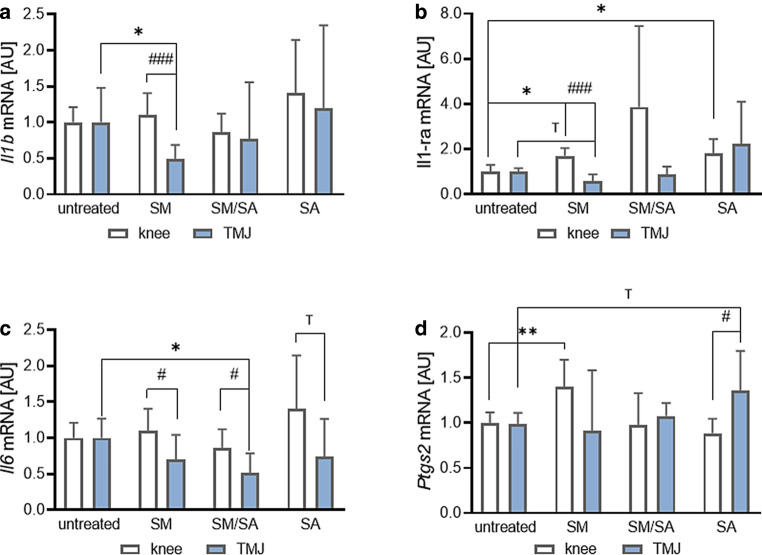
Effects of dynamic stretching protocols on the inflammatory genes *Il1b* (*n*_knee_ = 12, *n*_TMJ_ = 10; **a**), *Il1-ra* (*n*_knee_ = 8, *n*_TMJ_ = 7; **b**), *Il6* (*n*_knee_ = 12, *n*_TMJ_ = 8; **c**) and *Ptgs2* (*n*_knee_ = 12, *n*_TMJ_ = 11; **d**) in synovial fibroblasts derived from the knee and temporomandibular joint (TMJ). *AU* arbitrary unit, *SM* moderate, *SM/SA* intermediate, *SA* advanced. Statistics: Mixed-effects analysis with Geisser–Greenhouse correction followed by Holm–Sidak’s multiple comparisons tests. ^T^*p* < 0.10, *^,#^*p* < 0.05, **^,##^*p* < 0.01, ***^,###^*p* < 0.001 Auswirkungen von dynamischen Dehnungsprotokollen auf die Entzündungsgene *Il1b* (*n*_Knie_ = 12, *n*_TMJ_ = 10; **a**), *Il1ra* (*n*_Knie_ = 8, *n*_TMJ_ = 7; **b**), *Il6* (*n*_Knie_ = 12, *n*_TMJ_ = 8; **c**) und *Ptgs2* (*n*_Knie_ = 12, *n*_TMJ_ = 11; **d**) in synovialen Fibroblasten aus dem Knie- und dem Kiefergelenk (TMJ). *AU* arbiträre Einheit, *SM* moderat, *SM/SA* gemischt, *SA* anspruchsvoll. Statistik: Mixed-effects-Analyse mit Geisser-Greenhouse-Korrektur, gefolgt von Holm-Sidaks „multiple comparisons tests“. ^T^*p* < 0,10, ^*,#^*p* < 0,05, **^,##^*p* < 0,01, ***^,###^*p* < 0,001

Next, we investigated the expression of genes involved in inflammatory reactions. Interleukin-1-beta (Il1β) is a highly effective cytokine promoting the increase of neutrophil granulocytes and interleukin‑6. Surprisingly, we detected no changes in *Il1β* gene expression with any stretching protocol in knee-derived synovial fibroblasts (SM: *p* = 0.7274; SM/SA: *p* = 0.3952; SA: *p* = 0.1835). In TMJ-derived synovial fibroblasts moderate stretching even reduced *Il1β* gene expression (SM: *p* = 0.0274), while there were no changes with mixed or advanced stretching protocols (SM/SA: *p* = 0.7663; SA: *p* = 0.9358; Fig. 5a). With moderate stretching, we observed significant differences between synovial fibroblasts from the knee and the TMJ (SM: *p* < 0.001), while the other tested protocols revealed no effects (SM/SA: *p* = 0.9484; SA: *p* = 0.9484; Fig. 5a). Interleukin‑1 receptor antagonist (IL1RA) is an IL1β antagonist with inhibitory attributes to inflammation. As response to inflammation, IL1RA will be upregulated in order to dampen IL1β-mediated signalling [4, 58]. Both moderate (SM: *p* = 0.0104) and advanced stretching (SA: *p* = 0.0190) increased *Il1-ra* gene expression in knee-derived synovial fibroblasts, while moderate stretching had no effect (SM: *p* = 0.1330; Fig. 5b). In contrast to knee-derived synovial fibroblasts, cells from TMJ origin tended to reduce *Il1ra* expression with moderate dynamic stretching (*p* = 0.0982), while mixed (SM/SA: *p* = 0.7778) or advanced stretching (SA: *p* = 0.3892) had no effects. Moderate stretching revealed a significant difference between cells derived from the knee and the TMJ (*p* < 0.001; Fig. 5b). IL6 signalling is regulated by IL1β. Hence, we detected no changes in *Il6* gene expression in knee-derived synovial fibroblasts (SM: *p* = 0.7274; SM/SA: *p* = 0.3952; SA: *p* = 0.1835; Fig. 5c). In accordance, neither moderate (SM: *p* = 0.1921) nor advanced (SA: *p* = 0.5204) stretching affected *Il6* gene expression in TMJ-derived fibroblasts. Mixed stretching, however, resulted in a significant downregulation of *Il6* (*p* = 0.0302; Fig. 5c), indicating an anti-inflammatory reaction of TMJ-derived synovial fibroblasts. We observed differences in *Il6* gene expression in synovial fibroblasts depending on their origin after moderate (SM: *p* = 0.0382) and mixed stretching (SM/SA: *p* = 0.0303), while advanced stretching (SA: *p* = 0.0525) tended to downregulate *Il6* expression in the TMJ- compared to knee-derived fibroblasts (Fig. 5c). Prostaglandin endoperoxidase synthase 2 (PTGS2) is responsible for prostaglandin synthesis from arachidonic acid. In knee-derived synovial fibroblasts moderate stretching increased *Ptgs2* gene expression (*p* = 0.0032), while mixed (SM/SA: *p* = 0.9925) or advanced stretching (SA: *p* = 0.1124) had no effect (Fig. 5d). In contrast, advanced stretching tended to increase *Ptgs2* gene expression (*p* = 0.0998) in TMJ-derived fibroblasts with no effects of moderate (SM: *p* = 0.9680) or mixed stretching (SM/SA: *p* = 0.3773). Gene expression of *Ptgs2* upon advanced stretching was dependent on the origin of synovial fibroblasts (*p* = 0.0337; Fig. 5d). These data indicate that knee- and TMJ-derived synovial fibroblasts strongly differ in the gene expression profile of inflammatory genes in reaction to dynamic stretching protocols.

#### Impact on bone-remodelling gene expression

**Fig. 6:**
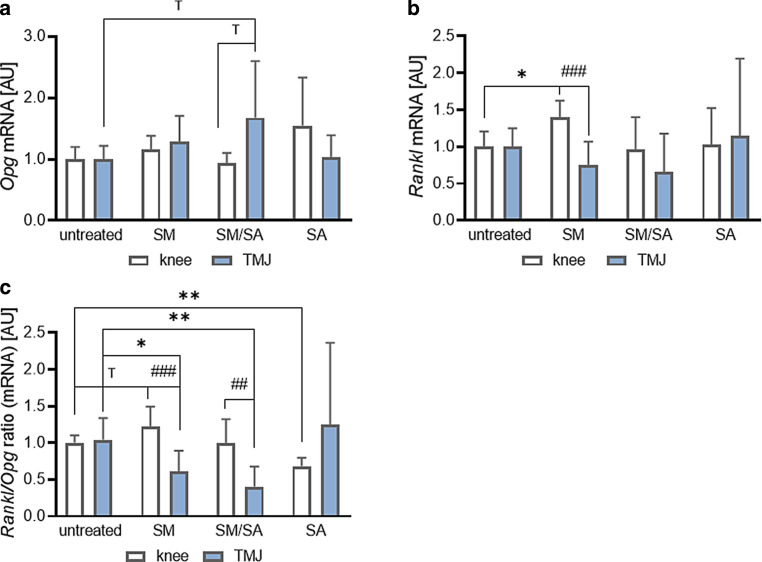
Effects of dynamic stretching protocols on the bone remodelling genes *Opg* (*n*_knee_ = 7, *n*_TMJ_ = 11; **a**) and *Rankl* (*n*_knee_ = 7, *n*_TMJ_ = 11; **b**) and the *Rankl/Opg *ratio (*n*_knee_ = 7, *n*_TMJ_ = 11; **c**) in synovial fibroblasts derived from the knee and temporomandibular joint (TMJ). *AU* arbitrary unit, *SM* moderate, *SM/SA* intermediate, *SA* advanced. Statistics: Mixed-effects analysis with Geisser–Greenhouse correction followed by Holm–Sidak’s multiple comparisons tests. ^T^*p* < 0.10, *^,#^*p* < 0.05, **^,##^*p* < 0.01, ^###^*p* < 0.001 Auswirkungen von dynamischen Dehnungsprotokollen auf die Knochenumbaugene *Opg* (*n*_Knie_ = 7, *n*_TMJ_ = 11; **a**) und *Rankl* (*n*_Knie_ = 7, *n*_TMJ_ = 11; **b**) sowie das *Rankl*/*Opg*-Verhältnis (*n*_Knie_ = 7, *n*_TMJ_ = 11; **c**) in synovialen Fibroblasten aus dem Knie- und dem Kiefergelenk (TMJ). *AU* arbiträre Einheit, *SM* moderat, *SM/SA* gemischt, *SA* anspruchsvoll. Statistik: Mixed-effects-Analyse mit Geisser-Greenhouse-Korrektur, gefolgt von Holm-Sidaks „multiple comparisons tests“. ^T^*p* < 0,10, ^*,#^*p* < 0,05, ^**,##^*p* < 0,01, ^###^*p* < 0,001

The expression ratio of receptor activator of NF-κB ligand (RANKL) to its decoy receptor osteoprotegerin (OPG) is a driving force for osteoclastogenesis [38]. In knee-derived synovial fibroblasts, none of the dynamic stretching protocols influenced *Opg* gene expression (SM: *p* = 0.2004; SM/SA: *p* = 0.7957; SA: *p* = 0.2319; Fig. 6a). Similarly, only mixed stretching tended to increase (SM/SA: *p* = 0.0970) *Opg* gene expression in TMJ-derived synovial fibroblasts with no effects of moderate (SM: *p* = 0.1630) or advanced stretching (SA: *p* = 0.9838). *Opg* tended to be differentially expressed in fibroblasts from the knee and the TMJ after mixed dynamic stretching (SM/SA: *p* = 0.0938; Fig. 6a). *Rankl* gene expression was elevated in knee-derived synovial fibroblasts after moderate stretching (SM: *p* = 0.0104), while neither mixed (SM/SA: *p* = 0.9933) nor advanced stretching (SA: *p* = 0.9966) affected *Rankl* gene expression (Fig. 6b). In contrast, TMJ-derived synovial fibroblasts did not change *Rankl* gene expression after any dynamic stretching protocol (SM: *p* = 0.1228; SM/SA: *p* = 0.2702; SA: *p* = 0.9433; Fig. 6b). Compared to knee-derived synovial fibroblasts, TMJ-derived cells reduced *Rankl* gene expression upon moderate stretching (SM: *p* < 0.001). *Rankl/Opg* ratio tended to be increased upon moderate loading (SM: *p* = 0.0791), while advanced stretching reduced the *Rankl/Opg* ratio significantly (SA: *p* = 0.0026; Fig. 6c) in knee synovial fibroblasts. In contrast, we observed a significant downregulation of the *Rankl/Opg* ratio in TMJ synovial fibroblasts with moderate (SM: *p* = 0.0164) and mixed stretching (SM/SA: *p* = 0.0017), while advanced stretching had no effect (SA: *p* = 0.8971; Fig. 6c). Thus, differences depending on synovial fibroblast origin seem to exist after moderate (SM: *p* < 0.001) and mixed (SM/SA: *p* = 0.0031) stretching (Fig. 6c), indicating different reactions to dynamic stretching.

## Discussion

Studies analysing the structural development of joints proved that it is difficult to define distinct determining factors of tissues and structures. Joint formation seems like an event of coincidence, guided by delicate processes of limb patterning, embryonic movement and extracellular matrix composition, while the right dose of local signalling events support the process of joint cavity formation [28, 35]. To further complicate this process, each joint needs its own factors of determination and develops from distinct germ layers, while the composition of structural components almost maintains the same. Focusing on already developed joints, we analysed synovial fibroblasts descended from two different layers to gain insight into osteoarthritis development and progression of the knee and temporomandibular joint (TMJ): synovial fibroblasts from the TMJ, descending from the ectoderm, and synovial fibroblasts from the knee joint, descending from the mesoderm. Although both synovial fibroblast populations are located in their corresponding synovial tissue and perform similar tasks in the upkeeping of joint homeostasis [30], we observed differences in their response to mechanical stress. Extracellular matrix remodelling processes play a role during embryogenic development, but also in the pathogenesis of joint disorders such as osteoarthritis (OA), which also affects the TMJ, and corresponding tissue infiltration [57].

Synovitis is an example of an arthritic event of tissue infiltration, as the synovial membrane experiences synovial lining hyperplasia, infiltration of macrophages and lymphocytes and neoangiogenesis, while the release of proteolytic factors favours cartilage breakdown [8, 33, 59] (for further information see review: [48]). While developing synovitis, synovial tissue experiences a pathological fibrotic change (synovial fibrosis), characterised by clinical symptoms of pain and stiffness, favoured by excessive extracellular matrix (ECM) deposition and mediated by synovial fibroblasts [25, 32, 45].

A factor regulating OA-induced cartilage degeneration is ADAMTS (a disintegrin and metalloproteinase with thrombospondin motif), capable of cleaving the major cartilage proteoglycan aggrecan [24, 40]. Also high levels of fragmented fibronectin indicate arthritic changes [5, 12]. Furthermore, a change of collagen content occurs during OA pathogenesis [39], while small fragments of hyaluronan support inflammatory processes [53]. In the context of ECM composition and remodelling, the markers tested in this study showed no different expression pattern between synovial fibroblasts derived from the knee compared to those derived from the TMJ. However, for certain types of mechanical strain, they differed in gene expression of *Col1a2* and *Adamts5*.

Also factors of inflammation experienced a differential expression, depending on the origin of the synovial fibroblasts. A study analysing topographically differing synovial fibroblast populations found distinct gene expression patterns for genes involved in limb patterning, chemotaxis, cell–cell interactions and inflammation [20]. Although the synovial fibroblasts tested were derived from the knee joint, hand and shoulder, we also were able to see differences in inflammatory gene expression pattern between synovial fibroblasts derived from the knee and the TMJ. The expression of *Ptgs2*, which is involved in prostaglandin E2 synthesis, supports cell proliferation events, as well as angiogenesis, apoptosis and immunoregulating processes, while being induced by cytokines, growth factors and inflammation [47, 56]. A PTGS2-inducing cytokine is IL-1β, acting via the ERK pathway [36]. Apart from IL-1β, also IL‑6 plays an arthritis- and inflammation-mediating role [11, 23]. In order to regulate initiated inflammatory events, IL‑6 and IL-1β stimulate the expression of Interleukin 1 receptor antagonist (IL1RA) [21]. By binding to interleukin 1 receptors, the endogenous negative-feedback regulator IL-1RA is able to reduce inflammation-induced signalling, while experiencing acute-phase protein attributes [21]. In our study, we observed an altered gene expression depending on origin of cells and exhaustion due to mechanical strain for all four inflammation-associated markers. While *Il-1ra* experienced an upregulation in gene expression under moderate stretching (SM) and advanced stretching (SA) conditions in the case of synovial fibroblasts derived from the knee, synovial fibroblasts derived from TMJ only experienced a trend in downregulating gene expression under low exhaustive conditions. This observation led to a clearly differing gene expression pattern based on synovial fibroblast descendance, which only was present in the moderate stretching condition. As *IL-1ra* acts responding to increased proinflammatory cytokine expression in order to regulate inflammatory events, we expected a similar gene expression for *Il1β* and *Il6*. While only *Il1β* gene expression was diminished for synovial fibroblasts derived from the TMJ during low exhaustive conditions, this was the only condition confirming a coherence between *Il1β* and *Il-1ra* gene expression.

A critical element of OA pathogenesis is bone resorption, mediating destructive and irreversible affliction, while increasing pain and decreasing mobility. The process of bone resorption is mediated by osteoclast formation and activation induced by receptor activator of nuclear factor κB ligand (RANKL). While the binding of RANKL to the RANK receptor induces bone resorption processes, the decoy receptor osteoprotegerin (OPG) has a higher binding affinity to RANKL and prevents RANKL-mediated signalling. Synovial fibroblasts act as principal RANKL-expressing cells, therefore playing a major role in bone destruction during arthritis [17]. In our experimental set-up, we only observed increased *Rankl* expression for low exhaustive conditions in synovial fibroblasts derived from the knee joint, while corresponding *Opg* expression was not altered. Therefore, *Opg* expression was only increased by mixed stretching conditions (SM/SA) for synovial fibroblasts derived from the TMJ. By assessing the *Rankl*/*Opg* ratio, insights into potential osteoclast activation and formation are obtained with a value higher than 1 indicating an imbalance in favour of *Rankl* activating osteoclast formation and a value smaller than 1 favouring *Opg*, thus diminishing osteoclast formation. For synovial fibroblasts derived from the knee joint, low exhaustive conditions favoured, while exhaustive conditions diminished the *Rankl*/*Opg* ratio. In contrast, low and intermediate exhaustive conditions had reducing effects on the *Rankl*/*Opg* ratio in synovial fibroblasts from the TMJ.

For all tested attributes (inflammation, ECM remodelling, bone remodelling), we saw similarities in gene expression behaviour and distinctly differing gene expression patterns between synovial fibroblast populations. As we compared the effect of mechanical strain to unstressed cells, we did not analyse the transcriptome of synovial fibroblasts originating from diverse tissues. However, we were able to see a distinctly different activation of gene expression between the tested cell populations. This shows that mechanical stress has a different impact on synovial fibroblasts and that synovial fibroblasts derived from different joints diverge in osteoclastogenesis and inflammatory activation. During embryonic development HOX genes (homeobox genes) define the head–tail axis and limb patterning by segmental expression and DNA transcription activation. However, when analysing gene expression in synovial fibroblasts of adult donors, distinct HOX gene expression is maintained corresponding to the anatomical origin [20]. Based on the differing gene expression of synovial fibroblast populations, HOX gene expression may influence inflammation via the NF-κB pathway [43, 52], while tissue-specific expression patterns of cell–cell interaction and inflammation [20] are boosted by mechanical strain. Our findings show that OA pathogenesis is a complex process with risk factors such as mechanical overuse vary in biasing OA dependent on the joint of origin.

## Conclusions

The responses in gene expression due to different dynamic stretching protocols are strongly dependent on the origin of synovial fibroblasts. Synovial fibroblasts derived from the temporomandibular joint reduce the *Rankl/Opg* gene expression ratio after moderate and mixed dynamic stretching, while, in contrast to genes involved in inflammation or bone remodelling, expression profiles of genes involved in extracellular matrix remodelling are mostly not affected by dynamic stretching independent of their origin. Our data indicate that risk factors for the development and progression of osteoarthritis such as mechanical overuse have a different pathological impact in the temporomandibular joint compared to the knee joint.

## Supplementary Information

**Supplemental Figure 1**: Analysis of expression of fibroblast specific genes (Cdh11, Cd248) and absence of marker genes for osteoblasts, macrophages, osteoclasts and myogenic cells (Bglap, Cd68, Itga7, MyoD1, Des) in primary cell cultures of knee synovial tissue explants (**a**) and TMJ-dissected synovial tissue (**b**)/**Ergänzende Abbildung 1**: Analyse der Expression von Fibroblasten-spezifischen Genen (Cdh11, Cd248) und Abwesenheit von Markergenen für Osteoblasten, Makrophagen, Osteoklasten und myogene Zellen (Bglap, Cd68, Itga7, MyoD1, Des) in primären Zellkulturen von Knie- (**a**) sowie von TMJ-synovialem Gewebe (**b**); **Supplemental Table 1**: Primers used for semiquantitative PCR analysis in murine synovial fibroblasts/**Ergänzende Tabelle 1**: Verwendete Primer für die semiquantitative PCR-Analyse in murinen synovialen Fibroblasten
