# Supplementary material for: Differential gene expression response of synovial fibroblasts from temporomandibular joints and knee joints to dynamic tensile stress
Source: J Orofac Orthop. 2021 Jun 17;83(6):361–75. doi: 10.1007/s00056-021-00309-y (PMC9596579; doi:10.1007/s00056-021-00309-y)
Supplement: Supplementary file 1 — Supplemental Figure 1: Analysis of expression of fibroblast specific genes (Cdh11, Cd248) and absence of marker genes for osteoblasts, macrophages, osteoclasts and myogenic cells (Bglap, Cd68, Itga7, MyoD1, Des) in primary cell cultures of knee synovial tissue explants (a) and TMJ-dissected synovial tissue (b)/Ergänzende Abbildung 1: Analyse der Expression von Fibroblasten-spezifischen Genen (Cdh11, Cd248) und Abwesenheit von Markergenen für Osteoblasten, Makrophagen, Osteoklasten und myogene Zellen (Bglap, Cd68, Itga7, MyoD1, Des) in primären Zellkulturen von Knie- (a) sowie von TMJ-synovialem Gewebe (b); Supplemental Table 1: Primers used for semiquantitative PCR analysis in murine synovial fibroblasts/Ergänzende Tabelle 1: Verwendete Primer für die semiquantitative PCR-Analyse in murinen synovialen Fibroblasten [file 56_2021_309_MOESM1_ESM.pdf]

Supplemental Figure 1 Analysis of expression of fibroblast specific genes (*Cdh11*, *Cd248*) and absence of marker genes for osteoblasts, macrophages, osteoclasts and myogenic cells (*Bglap*, *Cd68*, *Itga7*, *MyoD1*, *Des*) in primary cell cultures of knee synovial tissue explants (a) as well as TMJ-dissected synovial tissue (b)

Ergänzende Abbildung 1 Analyse der Expression von Fibroblasten-spezifischen Genen (Cdh11, Cd248) und Abwesenheit von Markergenen für Osteoblasten, Makrophagen, Osteoklasten und myogene Zellen (Bglap, Cd68, Itga7, MyoD1, Des) in primären Zellkulturen von Knie-(a) sowie von TMJ-synovialem Gewebe (b)

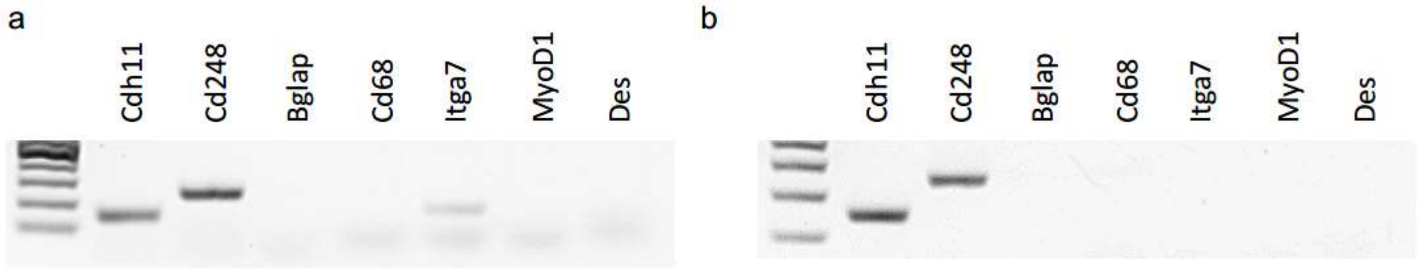

Supplemental Table 1 Primers used for semiquantitative PCR analysis in murine synovial fibroblasts

Ergänzende Tabelle 1 Verwendete Primer für die semiquantitative PCR-Analyse in murinen synovialen Fibroblasten

Gene symbol	Gene name	5'-forward primer-3'	5'-reverse primer-3'	Genebank accession number	Amplicon size, T_m, GC%
Cdh11	cadherin 11 [54]	AGGACGACACAGCCAATGGAC	AAGTCCTGCTTCTGCCGACTG	NM_009866.5	148 bp, 62°C, 57
Cd248	CD248 antigen, endosialin [26]	GCCAGCAGATGTGTGTCAA	GTAGGTGCCAGCCATAGGAT	NM_054042.2	238 bp, 58°C, 52-55
Bglap	bone gamma-carboxyglutamate protein [26]	GCTCTGTCTCTCTGACCTCACA	TAGATGCGTTTGTAGGCGG	NM_007541.3	231 bp, 61°C, 54-52
Cd68	CD68 antigen [26]	GCTTCTGCTGTGGAAATGC	GGTAGGTTGATTGTCGTCTGC	NM_001291058.1	234 bp, 59°C, 52
Itga7	integrin alpha 7	TTTGGGTTCTGTCAGCAGGGC	AGCTGGTCAGGGTCTGAGCTATC	NM_008398.2	137 bp, 63°C, 57-56
MyoD1	myogenic differentiation 1	ACTACAGTGGCGACTCAGATGC	GTGTCGTAGCCATTCTGCCG	NM_010866.2	109 bp, 62°C, 54-60
Des	desmin	AACTTCCGAGAAACCAGCCCC	GTTGCTGTGTAGCCTCGCTG	NM_010043.2	118 bp, 62°C, 57-60
